# Nanoceria: an innovative strategy for cancer treatment

**DOI:** 10.1007/s00018-023-04694-y

**Published:** 2023-01-19

**Authors:** Joyce L. Y. Tang, Shehzahdi S. Moonshi, Hang T. Ta

**Affiliations:** 1 Queensland Micro- and Nanotechnology Centre, Griffith University, Nathan, QLD 4111 Australia; 2 Bioscience Discipline Department, School of Environment and Science, Griffith University, Nathan Campus, Brisbane, QLD 4111 Australia; 3 Australian Institute for Bioengineering and Nanotechnology, University of Queensland, St Lucia, QLD 4072 Australia

**Keywords:** Nanomedicine, Cerium oxide, Redox-responsive nanozymes, Oxidative stress, Tumour hypoxia, Treatment development

## Abstract

Nanoceria or cerium oxide nanoparticles characterised by the co-existing of Ce^3+^ and Ce^4+^ that allows self-regenerative, redox-responsive dual-catalytic activities, have attracted interest as an innovative approach to treating cancer. Depending on surface characteristics and immediate environment, nanoceria exerts either anti- or pro-oxidative effects which regulate reactive oxygen species (ROS) levels in biological systems. Nanoceria mimics ROS-related enzymes that protect normal cells at physiological pH from oxidative stress and induce ROS production in the slightly acidic tumour microenvironment to trigger cancer cell death. Nanoceria as nanozymes also generates molecular oxygen that relieves tumour hypoxia, leading to tumour cell sensitisation to improve therapeutic outcomes of photodynamic (PDT), photothermal (PTT) and radiation (RT), targeted and chemotherapies. Nanoceria has been engineered as a nanocarrier to improve drug delivery or in combination with other drugs to produce synergistic anti-cancer effects. Despite reported preclinical successes, there are still knowledge gaps arising from the inadequate number of studies reporting findings based on physiologically relevant disease models that accurately represent the complexities of cancer. This review discusses the dual-catalytic activities of nanoceria responding to pH and oxygen tension gradient in tumour microenvironment, highlights the recent nanoceria-based platforms reported to be feasible direct and indirect anti-cancer agents with protective effects on healthy tissues, and finally addresses the challenges in clinical translation of nanoceria based therapeutics.

## Introduction

Cancer is the general term utilised to demonstrate a heterogenous group of diverse diseases characterised by rapid, uncontrolled growth of abnormal cells that can affect any part of the body. Cancer can metastasize and spread to other organs if not detected at an early stage, making it a difficult disease to treat, resulting in a leading cause of death globally [1]. The World Health Organisation’s International Agency for Research (WHO IARC) https://gco.iarc.fr/today/online-analysis-pie estimated 19.3 million cancer diagnosis with more than 50% mortality (9.96 million) in 2020. Despite advancements in cancer treatment strategies, cancers including lung, colorectal, prostate and stomach cancers still demonstrate poor five-year survival rate [2] especially with the COVID-19 global pandemic delaying diagnosis and disrupting access to cancer care and treatment options [3, 4]. Factors contributing to poor prognosis and cancer relapses including metastasis, intrinsic and acquired resistance to chemotherapeutic drugs revealed the ongoing need to enhance conventional therapies such as chemo- and radiation therapies, and to discover new, and innovative treatment approaches [5, 6]. One popular strategy is the engineering of redox-sensitive nanomaterials with the capability of regulating reactive oxygen species (ROS) in biological systems for desired treatment outcomes [7].

ROS including superoxide anions (O_2_^−^), hydrogen peroxide (H_2_O_2_), hydroxide radicals (^·^OH) and singlet oxygen (^1^O_2_) are crucial for signal transduction and cell survival [8]. ROS levels in cells are tightly regulated by antioxidant enzymes for redox homeostasis. Consequently, regulated ROS can promote both cell damage and cell survival and has been shown to be biologically important in autophagy, immune cell function, stem cell differentiation, cellular proliferation, and adaptation to hypoxia [9]. ROS was found to be associated with cancer, initially revealed by the elevated levels of ROS production in human tumour cells [10]. Further studies suggested that ROS have a dual role in cancer tumorigenesis and cancer cell survival via: (1) increased ROS production promoting pro-tumourigenic signalling events to facilitate cancer cell proliferation, metastasis, angiogenesis and adapting to hypoxia resulting in aggressive tumour growth; (2) reinforced ROS depletion to maintain ROS homeostasis below the toxic threshold [11]. Cancer cells adapting to tumour microenvironment (TME) have increased basal ROS level with a higher rate of both ROS production and ROS scavenging in comparison to healthy cells, which potentially increase their susceptibility to ROS or redox manipulation therapies [12]. Thus, enhancing ROS production and/or inhibiting ROS elimination to force excessive accumulation in tumours are feasible strategies as effective cancer therapies [7, 13, 14]. Several chemotherapeutics have been shown to increase ROS levels in cancer cells over the survival threshold and trigger oxidative stress-induced cancer cell death [13, 15]. Established anti-cancer drugs such as paclitaxel [16], doxorubicin [17] and cisplatin [18] promoted the accumulation of ROS beyond the tolerability threshold which contributed to their potent cytotoxicity effects. Cancer metastatic progression is a complex process consists of a series of sequential steps including cancer cells dissociation from a primary tumour, intravasation into and survival in the circulation, arrest in visceral organs, adhesion to endothelial cells, extravasation into tissues, proliferation and vascularisation of the metastatic lesions [19]. Throughout the metastatic progression, sublethal levels of ROS had been shown to be involved in activating/increasing the expression of various molecules closely related to the formation of metastatic colonies and angiogenesis including metalloproteinases, adhesion molecules, epidermal growth factor (EGF), EGF receptor and vascular endothelial growth factor (VEGF) [19]. Therefore, pro-oxidant therapeutic approaches that exacerbate oxidative stress on cancer cells could inhibit occurrence of distant metastasis not only through limiting cell survival, but also decreasing expression of pro-metastatic biomolecules that cancer cells interact with to promote metastatic progression [19, 20].

Nanoparticles (NPs) have been widely researched for use in medicine due to two key physicochemical properties: (1) high surface-to-volume ratios potentially increasing efficacy while decreasing biotoxicity; (2) high capabilities for multi-functionalisation especially through the incorporation of targeting ligands specific to disease types [21–23]. Nanomaterials can either exert cancer-killing effects themselves or are fabricated as a hybrid nanosystem, comprising different functionalities such as nanocarrier, therapeutic and imaging agent to address the heterogeneity and complexity of TME [24, 25]. The use of NPs carrier can protect the therapeutic drug from chemical and enzymatic degradation and premature clearance, allowing sufficient drug dosage to reach tumour sites [26–28]. ROS-based NPs such as nanozymes, synthesised with intrinsic ROS generation and/or depletion properties can directly induce oxidative stress on tumour cells or sensitise cancerous tissues to induce desired therapeutic outcomes [29–31]. ROS-responsive nanoplatform also allows controlled and targeted delivery of drugs to tumour sites, whilst ensuring stability, plasma half-life and bioavailability of the drugs administered [32].

**Fig. 1:**
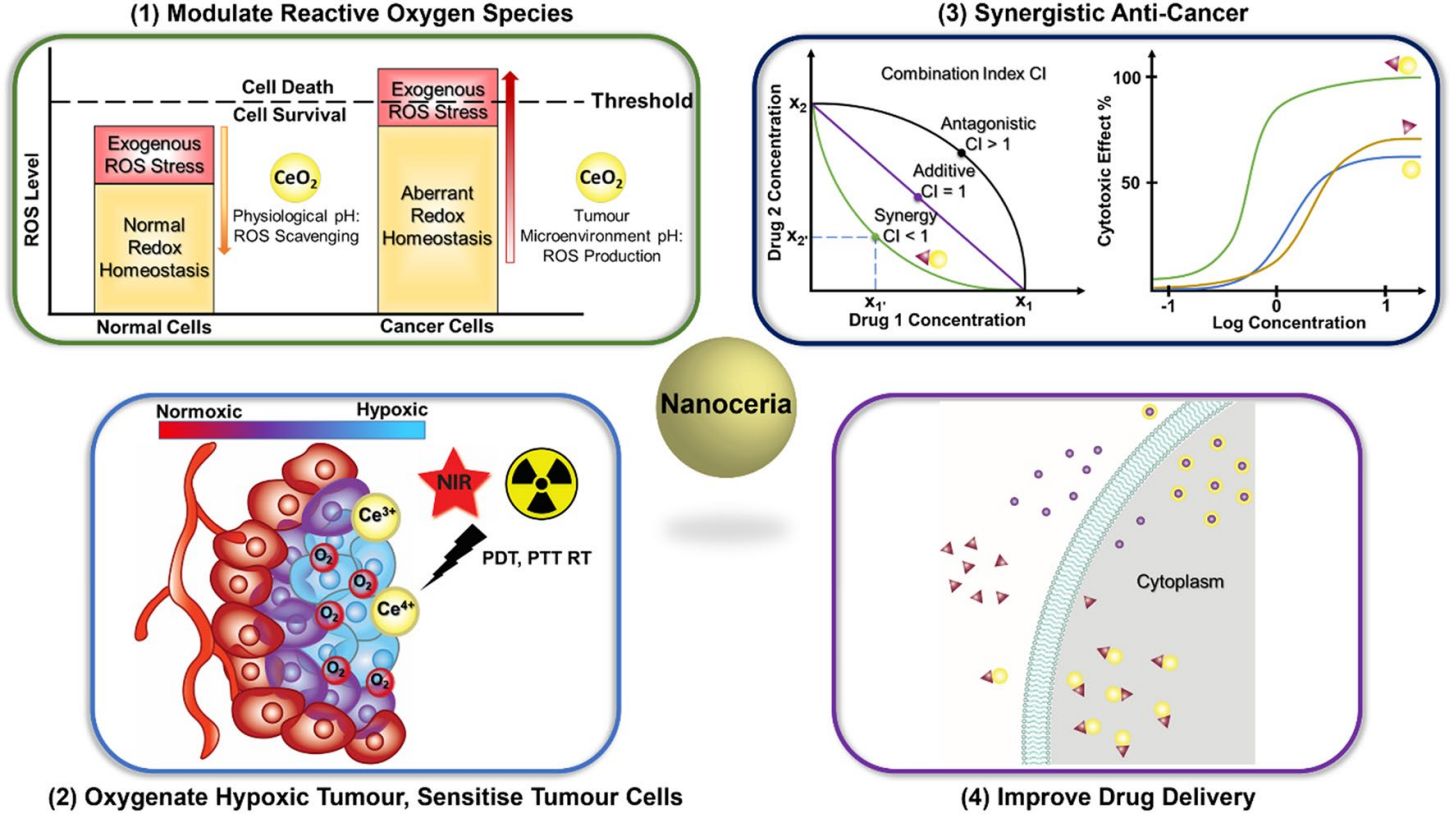
Applications of nanoceria in cancer treatment. (1) Protect normal cells from oxidative stress through ROS scavenging activities at physiological pH; induce oxidative stress on cancer cells through ROS production at slightly acidic tumour microenvironment. (2) Generate molecular oxygen to oxygenate hypoxic tumour and sensitise tumour cells for photodynamic, photothermal and radiation therapies. (3) Enhance cancer killing through synergistic combination with other anti-cancer agents. (4) Act as nanocarriers to improve delivery of anti-cancer drugs

Cerium oxide NPs or nanoceria have then been taken into consideration as potential anti-cancer agent due to the unique chemistry of cerium oxide [33, 34]. Cerium is the most abundant element in the lanthanide series which is unique from other lanthanoid rare earth metals that exhibit in the trivalent state, as it can exist in two oxidation states: fully reduced trivalent cerous Ce^3+^ and fully oxidised tetravalent ceric Ce^4+^ [35, 36]. Cerous Ce^3+^ and ceric Ce^4+^ atoms co-exist on the lattice surface of nanoceria, presenting oxygen vacancies for reduction–oxidation reactions [35–37]. Cerium oxide NPs are utilised as nanozymes, mimicking the catalytic activity of redox enzymes such as superoxide dismutase (SOD), catalase, phosphatase, oxidase peroxidase, and phosphotriesterase, advantaging from their ability to rapidly convert between valency states [38]. The non-stoichiometry ratio of Ce^3+^/Ce^4+^ on the surface determines the catalytic performance of the NPs and is shown to be affected by synthesis methods [38–40]. Cerium can easily alternate and adjust its electronic configuration responding to the immediate microenvironment. This ability allows nanoceria to manifest either antioxidant or pro-oxidant activities [33, 34, 41–43]. Its anti-oxidative effects have been employed in the development of numerous cerium oxide-based nanomaterials for ROS-related diseases [39, 42, 44, 45]. Studies in the anti-cancer potential of nanoceria emerged in recent years exploring its pro-oxidative effects inducing cancer cell death whilst the antioxidant properties suppressing tumour growth and protecting normal tissues from oxidative stress. Nanoceria has demonstrated significant therapeutic implications, either modulating ROS to kill or sensitise tumour cells while protecting neighbouring tissues or as a drug carrier to improve treatment outcomes and patient’s prognosis (Fig. 1). A comprehensive review of the biomedical applications of nanoceria was published in 2021, covering a wide range of biological properties with a brief discussion on the anti-cancer activities of nanoceria [46]. Another review on a similar topic was published recently focusing on the use of nanoceria as biosensors in cancer diagnosis and as a chemical sensitiser for cancer therapies with limited coverage on cancer-killing properties of nanoceria reported in the last 3 years [47]. The most recent summary of nanoceria’s anti-cancer effects was a perspective article published in 2018 discussing the multifaceted activity of nanoceria in the prevention and treatment of cancer [48]. Therefore, our comprehensive review aims to summarise recent research on nanoceria in cancer treatment, focusing on the NPs’ interventions in the adapted tumour redox environment.

## Dual-catalytic activities

**Fig. 2:**
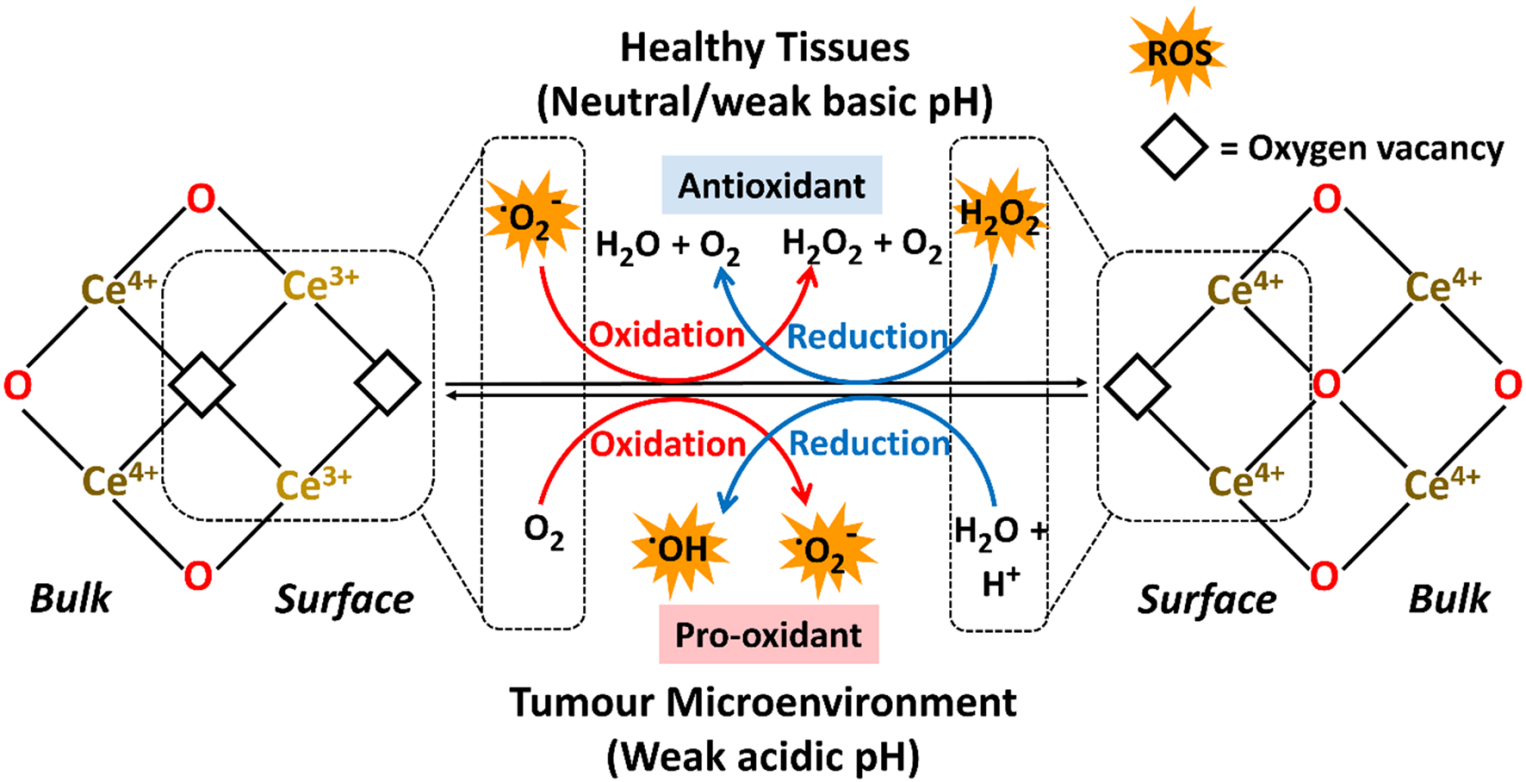
Nanoceria or cerium oxide nanoparticles are characterised as the co-existence of Ce^3+^ and Ce^4+^ with oxygen vacancies on the surface lattice. Depending on the immediate environment, nanoceria exerts antioxidative activities at neutral pH that scavenges reactive oxygen species (ROS) within healthy tissues whilst acting as pro-oxidant producing ROS at the weak acidic tumour microenvironment

The co-existing Ce^3+^ and Ce^4+^ on nanoceria make it self-sufficiently dual-catalytic, promoting both anti-oxidation and oxidation processes. The catalytic activities of nanoceria as nanozymes can be tuned at the synthesis steps, adjusting key physicochemical properties such as size, morphology, hetero-atom doping and surface modification [40, 49, 50]. The nanoceria crystals can be engineered to have different facet exposure and surface microstructures through varying synthesis conditions (amount of sodium hydroxide, reaction temperature, etc.) [51, 52]. This resulted in nanoceria of different morphologies and size: nanooctahedra (dominant [111] planes exposed), nanocubes (predominantly [100] surface facets) or nanorods ([100] and [111] or [110] planes exposed depending on synthetic procedures) [53], and that nanorods demonstrated highest peroxidase-like activity due to having the most abundant defects and richest oxygen vacancies (Ce^3+^ dominating) detected on the surfaces [52]. This finding was further supported by Fu et al. where the authors reported the preparation of nanoceria with various morphologies using a one-step hydrothermal synthesis method. It was reported that rod shape nanoceria had a substantially higher percentage of Ce^3+^ (66.6%) over other morphology formulations (up to 26.8%) [54]. Doping with cations such as Al^3+^ impede the surface diffusion of Ce ions, forming –Al–O–Ce–O– clusters that significantly improved the reversible oxygen storage capacity of nanoceria [55]. Once applied in biological systems, the bioactive properties of nanoceria are generally determined from Ce^3+^/Ce^4+^ ratio that are affected by the surrounding microenvironment, with pH and oxygen tensions being the pivotal factors (Fig. 2) [56].

### Ce^3+^/Ce^4+^

Nanoceria is characterised by the co-existence of fully oxidised Ce^4+^ and fully reduced Ce^3+^. The Ce^3+^/Ce^4+^ ratio presented on a surface lattice is instrumental in determining the catalytic properties of Ce. When Ce^3+^ is dominating, the NPs exhibit higher SOD-like activity that catalyses transformation of superoxide radicals to hydrogen peroxides and oxygen: O^·^_2_^-^ + Ce^4+^ → O_2_ + Ce^3+^ and O^·^_2_^-^ + Ce^3+^ + 2H^+^ → H_2_O_2_ + Ce^4+^ [57]. When Ce^3+^/Ce^4+^ ratio is low, higher catalase- and phosphatase-mimetic activities catalysing the degradation of H_2_O_2_ and phosphoric acid monoesters, respectively, are observed eg. H_2_O_2_ + 2Ce^4+^ → 2H^+^ + O_2_ + 2Ce^3+^ and H_2_O_2_ + 2Ce^3+^ + 2H^+^ → 2H_2_O + 2Ce^4+^ [37, 58]. Doping of lower valence cations in nanoceria has been shown to improve Ce^3+^/Ce^4+^ ratio, creating more oxygen vacancies and thus obtaining higher catalytic activity, decomposing H_2_O_2_ to ·OH. Among doped metals including Cu, Fe (Fe^2+^ and Fe^3+^), Co, Zn and Mn, Cu-doped cerium oxide demonstrated the largest fraction of Ce^3+^ (Ce^3+^/Ce^4+^ ratio Cu-doped vs naive: 0.334 vs 0.175) and highest catalytic capacity in the production of hydroxyl radicals. Advantaged from the elevated Ce^3+^ on the ceria lattice surface, near complete depletion of H_2_O_2_ was reported. The enzyme-mimicking activity of Cu-CeO_2_ was found to be higher than CeO_2_ evident by the stronger ·OH signals detected in the electron spin spectroscopy (ESR) spectrum [59]. Whilst many studies had showed that surface CeO_2_ acts as a catalytically active site and Ce^3+^/Ce^4+^ ratio is highly correlated to the enzymatic activity of nanoceria, researchers should always experimentally verify the enzyme-mimicking activities of the engineered materials instead of making assumptions based on the measured ratio. A recent study reporting nanoceria with varying morphologies as miRNA delivery platform demonstrated that octahedral and cubic nanoceria did show a positive correlation between Ce^3+^/Ce^4+^ with SOD and CAT activities [54]. Interestingly, the CeO_2_ nanorod with the highest Ce^3+^ among all formulations was found to have the lowest enzymatic activity for both SOD and CAT mimic activities [54]. The authors suspected that the CePO_4_ regions from the low concentration of Na_3_PO_4_ introduced as a mineraliser and shape control additive in the synthesis of nanorod increased the level of calculated Ce^3+^ and could diminish enzymatic activity [54].

### pH-responsive

The oxidation states of nanoceria are shown to be pH-responsive with higher efficacy of ROS production in more acidic conditions (pH 5.4 > pH 6.5 > pH 7.4 [60]) and vice versa for ROS scavenging [61, 62]. Hence, the difference in acid–base status between normal and tumour tissues can be exploited and is instrumental in anticancer drug design and discovery [63]. Normal tissues interstitial pH are mostly around 7.2–7.5 under well-perfused conditions. In contrast, TME is characterised to be slightly acidic at pH 6.4–7.0 with value as low as 5.6 reported. This is due to the uncontrolled cell proliferation and high metabolic activity of tumours accompanied by insufficient blood perfusion which leads to inefficient removal of acidic metabolic waste [64–66]. Interestingly, a study demonstrated by Liu et al., whereby bare nanoenzyme cerium oxide displayed weaker catalase-like activity at pH 5.5 in comparison to a higher pH of 7.4 [67]. Nonetheless, upon encapsulation in a metal–organic framework core–shell nanohybrid system, a lower pH resulted in the conversion of H_2_O_2_ into highly cytotoxic ·OH radicals that kill tumour cells with a significant increase in radicals generated when pH value decreased from 6.6 to 6.2. This study established that the encapsulation and protection of the cerium oxide core with the metal shell resulted in a ninefold higher apoptotic efficiency in hypoxic cells than that without the shell and thus stimulating a self-feedback system through caspase-3 initiation [49, 68]. Results from a recent study demonstrated that HeLa and U87MG cancer cells exhibited > 80% cell viability after treatment at neutral pH but ~ 40% or less cells were viable at pH ≤ 6.6, confirming enhanced toxicity in a lower pH environment [68]. The ROS scavenging/ROS generating capability under irradiation conditions of nanoceria is also pH-sensitive. Upon X-ray irradiation (total dose 5 Gy, 1 Gy min^−1^), a dextran-coated gadolinium-doped nanoceria Ce_0.9_Gd_0.1_O_1.95_ generated ROS in pH 6.0 Tris buffer but scavenged ROS, almost halving the hydrogen peroxide level in pH 8.0 Tris buffer [69].

Despite the general understanding that the oxidation states, Ce^3+^/Ce^4+^ ratio and pH environment critically affect the redox reactions of nanoceria, very few studies provided clarification of Ce^3+^/Ce^4+^ on the synthesised materials. The pH-responsive activities were generally assessed in cell-free assays under controlled experimental conditions, lacking the complexity and heterogeneity of physiological environments. In addition to that, there are also limited research into the oxidation states of nanoceria when the nanoparticles enter cells and reside in tissues. Szymanski first demonstrated in 2016 that a shift to a higher Ce^3+^/Ce^4+^ was detected when nanoceria entered the cells, indicating that there was a net reduction of nanoceria in the intracellular environment [70]. A similar ratio was found in the cytoplasm and lysosomes, suggesting that the net reduction of nanoceria was an early event within the internalisation pathway [70]. Unfortunately, this was the only study that reported characterisation of nanoceria status in organelles involved in internalisation.

### Oxygen tension responsive

Hypoxia, defined as “a condition of low oxygen tension”, with O_2_ level typically in the range of 1–5% in comparison to normoxia sitting at 10–21% [71], is commonly found in solid tumours due to poor vascularisation in response to the unregulated proliferation and metabolism of cells starving endogenous oxygen supply [72]. Hypoxic condition is often linked to cancer progression, metastasis [73], immunosuppression [74], and limited response [75] or resistance to treatment [76], leading to poor clinical outcomes. Under the hypoxic and acidic TME, nanoceria acted as pro-oxidant to inhibit the induction of hypoxia-inducible factor 1-alpha (HIF-1α) and the subsequent oncogenic signalling pathways [77]. It was reported that HIF-1α level decreased gradually over time after treatment with a 2D graphdiyne GDY–CeO2 nanocomposites under both normoxic and hypoxic conditions. Nonetheless, these nanocomposites sensitized both Herceptin-sensitive and Herceptin-resistant cells whereby Herceptin treatment resulted in more potent cytotoxic effects in cancer cells under hypoxia than under normoxia [78]. H_2_O_2_, a relatively stable ROS, is largely produced by human tumour cells without exogenous stimulation [10]. When exposed to excess level of H_2_O_2_, nanoceria primarily mimicked catalase activity, increasing production of O_2_ over time, in contrast to no changes in O_2_ level detected when H_2_O_2_ was not supplied. The generation of molecular O_2_ plays a key role in relieving tumour hypoxia and sensitising cancerous cells for enhanced therapeutic responses [67, 79].

Consequently, nanoceria is capable of targeting the “Triad of Death” in cancer, which consists of primary tumour growth, drug resistance and metastasis [80], through its catalytic activities that reprogram the aberrant redox environment in cancers: production of cytotoxic ROS to kill cancer cells, inhibit tumour growth and suppress the expression of pro-metastatic molecules; production of O_2_ to sensitise treatment-resistant cells and relieving hypoxic conditions to inhibit oncogenic signalling cascades associated with progression to metastatic disease.

## Nanoceria as potential cancer therapeutic agent

The unique properties of nanoceria that favours as an oxidising agent under acidic and hypoxic conditions, which are both key features of aggressive tumour progression, while acting as an antioxidant under physiological conditions to protect normal cells presents it as an attractive anti-cancer agent. Recent publications reporting the cancer-killing effects of nanoceria are summarised in Table 1.

**Table 1:** List of nanoceria reported to exert anti-cancer effects in 2019–2021

Nanoplatform	Size, zeta potential, morphology	Targeting moieties on NPs	Cell lines	In vitro	In vivo/ex vivo
Nanoceria [63, 81]	34.1 nm − 32.9 mV Cubic fluorite	–	Murine fibrosarcoma WEHI164 cells Normal fibroblast L929 cells	Significant cytotoxic effects on WEHI164 cells from 15.63 μg/mL ROS production in WEHI164 cells, ROS scavenging in L929 cells Number of apoptotic and necrotic WEHI164 cells increased Pro-apoptotic Bax mRNA expression upregulated in WEHI164 cells, downregulated in L929 cells	0.5 mg/kg in 100 μL PBS injected intraperitoneally twice a week for four weeks in WEHI164 tumour-bearing mice Inhibition of tumour growth Selectively accumulated in tumour No significant effects on liver and kidney functions Pro-apoptotic Bax expression upregulated, anti-apoptotic Bcl2 expression downregulated in tumours Higher number of TUNEL-positive cells per unit area in nanoceria-treated tumours indicating increased numerical density of apoptotic tumour cells
CeO_2_-GO_x_@CCM [68]	~ 50 nm − 20.43 mV Cubic	Coated with HeLa cancer cell membrane (CCM) for homotypic targeting	Cervical cancer HeLa cells Human umbilical vein endothelial cells HUVEC	Preferential accumulation in HeLa cells Increased ROS production in HeLa cells at slightly acidic pH ≤ 6.6 No obvious ROS production in HUVEC cells at pH 7.4	10 mg/kg in 200 μL PBS injected intravenously in HeLa tumour-bearing mice Significantly higher accumulation in tumour Complete tumour growth suppression with mice weight remained stable for 14 days High level of tumour cell apoptosis and necrosis observed in H&E stained tumour tissue No significant morphological changes observed in H&E-stained major organs
Ce_0.9_Gd_0.1_O_1.95_ [69]	4–6 nm Cubic	–	Breast cancer MCF-7 cells Human mesenchymal stem cells hMSC	Predominantly localised in cytoplasm and lysosomes No decrease in hMSC viability with the absence of apoptotic cells; dose-dependent decrease in viability and increase in number of apoptotic cells for MCF-7 Significantly reduced MTMP of MCF-7 cells exposed to NPs at 2.5 and 5 mg/mL Significantly increased expression levels of proapoptotic CD40 gene and genes related to antioxidant defence processes indicating activation of oxidative stress No significant haemolytic activity in the absence of serum proteins	
DOX loaded HA-Cu-CeO_2_@CCM [59]	–Ce^3+^/Ce^4+^ ratio: 0.334	Coated with MDA-MB-231 cancer cell membrane (CCM) for homotypic targeting	Breast cancer MDA-MB-231 cells Mouse myoblast C2C12 cells	Significantly higher uptake in source cancer cells Excellent hemocompatibility Near 100% killing of cancer cells resulted from the synergistic effect of DOX and Ce-CeO_2_ Depleted DOX-induced ROS level and increased viability of C2C12 cells	5 mg/mL in 200 μL PBS injected intravenously in MDA-MB-231 tumour-bearing mice Near complete tumour growth inhibition (98.5%) Dramatically elevated signal of superoxide indicator dihydroethidium (DHE) staining in treated tumour Down-regulated expression of proliferative marker Ki67 in the treated group H&E and TUNEL staining significantly higher proportion of DNA damage and apoptosis in tumour tissues Blood routine examination, liver and kidney function indicators and myocardium damage indicator were all in a normal range No pathological changes and significant damages in major organs observed indicating potential to relieve DOX-induced systemic toxicity
Green synthesised CeO_2_ [105]	35–40 nm	–	HeLa cells	Dose-dependent cytotoxicity on HeLa cells when used at higher doses (50–125 μg/mL) Cell morphology changed with more apoptotic HeLa cells observed at 100 μg/mL	–
CeO_2_ [12]	1–10 nm	–	Melanoma A375 cells Normal human epidermal melanocytes NHEM	Higher basal ROS level in A375 cells that was even higher upon nanoceria treatment High level of mitochondrial O_2_^−^ detected in A375 cells potentially the source for SOD-like activity of nanoceria producing H_2_O_2_ Loss of MTMP induced in A375 cells Number of intermediate (0.5–5 μm) and fragmented (< 0.5 μm) mitochondria increased in treated A375 cells Cell viability significantly decreased in A375 cells but preincubation with PEG-catalase fully restored the viability of treated A375 cells	–
HA-CePEI-NPs [85]	70 nm − 20 mV Spherical	Conjugated with hyaluronic acid HA to target CD44 receptor	MDA-MB-231 Human breast epithelial HBL-100 cells	Significant concentration-dependent cytotoxicity in cancer cells Loss of MTMP in cancer cells ROS levels inversely correlated with GSH levels Significantly declined Bcl-2 and elevated cytosolic Cyt c levels Dose-dependent activation of caspases-3 and -9	–
CeO_2_ [83]	30–40 nm − 4.9 mV Cubic fluorite	–	Colorectal carcinoma HCT116 cells Human embryonic kidney HEK293 cells	Significantly lower IC50 for HCT116 (50.48 μg/mL) than HEK293 (92.03 μg/mL) Dose-dependent increased ROS production in HCT116 Cell population in early and late apoptosis significantly increased Enhanced pro-apoptotic Bax, Bak, Cyt c, decreased antiapoptotic Bcl2 Increased activation of caspases-3 and -9 Increased number of phosphorylated histones γ-H2AX foci Suppressed expression of Mdm2 Increased phosphorylation of p53, activation of p21 Increased DNA fragmentation	–
CeO_2_ [86]	12 nm − 14 mV Spherical Ce^3+^/Ce^4+^ ratio: 0.94	–	Hepatocellular carcinoma HepG2 cells	Endocytic uptake and retention of NPs	0.1 mg/kg in 500 μL saline solution injected intravenously in rats induced with hepatocellular carcinoma (HCC) Development of HCC nodules attenuated in the liver of treated rats Significant increase in TUNEL-positive cells in the liver of treated rats Lower macrophage infiltration measured by CD68 staining, decreased number of Ki67-positive cells in the treated group Level of phosphorylated extracellular signal-regulated kinase 1/2 (P-ERK1/2) decreased Level of linoleic acid that contributed to HCC development reversed in treated group Overall survival comparable to a group treated with tyrosine kinase inhibitor sorafenib
PN-CeO_2_-PSS [66]	Porous nanorod: ~ 60.0 nm length, ~ 8.0 nm diameter, ~ 2.0–4.0 nm pore size; PN-CeO_2_-PSS—70 kDa: 306.5 nm	–	HepG2 Normal human hepatic stellate LX-2 cells	Non-toxic to LX-2 cells at 250 μg/mL Dose-dependent cytotoxicity, increased caspase-3 expression in HepG2 cells under mild acidic conditions (pH 6.5) Apoptotic body, chromatin condensation, nucleic fragmentation and membranolysis observed in treated HepG2 cells Enhanced ROS and malonaldehyde (MDA) levels at pH 6.5	5 mg/mL in 100 μL saline solution injected intravenously in HepG2 tumour-bearing mice H&E staining showed significant necrosis and apoptosis in treated tumour tissues Significant DHE staining signal observed indicating increased ROS generation in nanoceria-treated tumours Near complete inhibition (96.1%) of tumour growth Higher rate of tumour tissue apoptosis in TUNEL assay No significant toxicity to major organs No significant effects on blood biochemical indexes and liver, kidney function parameters No significant haemolysis

### In vitro studies

Several studies have pinpointed that nanoceria exhibits anti-cancer properties through the activation of p53-dependent, mitochondrial-mediated, oxidative stress-triggered apoptosis. Nourmohammadi et al. revealed that nanoceria prepared by the co-precipitation method exerted enhanced cytotoxic effects, ROS modulation and apoptotic genes expression in fibrosarcoma cells in comparison to normal fibroblast [63, 81]. Adebayo et al. proposed that a polyacrylic acid polymer-coated nanoceria is a potential antitumorigenic agent by targeting cellular pathways linked to oxidative stress, inflammation, and apoptosis [82]. Cu-doped nanoceria with enhanced hydroxyl radicals production capability acted as pro-oxidant in cancer cells, which elevated intracellular ROS levels, resulting in a high proportion of DNA damage [59], and triggering the p53-dependent mitochondrial signalling pathway leading to cellular apoptosis. Datta reported the enhanced number of phosphorylated histone γ-H2AX, a marker of DNA damage, which then activated tumour suppressor p53 and disrupted ubiquitin ligase Mdm2 binding to p53 protein [83]. The increased number of phosphorylated p53 also elicited activation of its transcriptional target p21 that led to G1 or G2/M cell cycle arrest [83, 84]. Aplak et al*.* showed that melanoma A375 cells exhibited a significantly higher basal ROS level than their physiological counterpart NHEM cells making them more susceptible to additional oxidative provocation. The H_2_O_2_ IC_50_ value for NHEM cells (250.6 μM) was ~ 3.5 times more than that of A375 cells (72.5 μM). High level of superoxide was detected in the mitochondria of A375 cells, which became the substrate for nanoceria SOD-mimetic activity producing H_2_O_2_ [12]. The increased H_2_O_2_ production was reported to cause the loss of mitochondrial membrane potential (MTMP) that disrupted mitochondrial structure and functions, and ultimately led to cancer cell death [12, 69]. The oxidative-stress-induced cell death was supported by the findings in which preincubation of cells with catalase that catalysed decomposition of H_2_O_2_ into non-toxic H_2_O and O_2_ fully rescued the cells from nanoceria-induced cytotoxicity [12]. Excess intracellular ROS depleted glutathione (GSH), depolarised mitochondrial membrane and decreased level of antiapoptotic Bcl-2 that regulates the permeability of the mitochondrial outer membrane and inhibits the release of cytochrome c (Cyt c) [83, 85]. Increased level of pro-apoptotic Bax and Bak in cytosol will be recruited to the mitochondria following stress signalling which then released Cyt c from mitochondria into cytosol [83]. Increased cytosol Cyt c level caused the activation of caspases-3 and -9 which ultimately induced programmed cell death of cancer cells [83, 85]. MDA-MB-231 cells treated with hyaluronic acid tagged nanoceria developed using oxidation method with poly(ethylenimine (PEI) showed irreversible nuclear chromatin condensation (pyknosis) and nuclear fragmentation, indicating the occurrence of nuclear apoptosis. Increase in the population of cells in the G2/M phase was also reported, revealing that nanoceria not only activated intrinsic apoptotic pathway but also reduced cell survival by preventing cells from entering mitosis and enabling the repair of DNA damage [85]. Popov et al*.* analysed the effects of an ultra-small dextran-coated gadolinium-doped nanoceria Ce_0.9_Gd_0.1_O_1.95_ on the gene expression of cancer and normal cells. Increased transcriptional activity of proapoptotic CD40 genes and a panel of antioxidant defence genes indicated activation of oxidative stress. As opposed to cancer cells, normal cells (human mesenchymal stem cells hMSCs) treated with nanoceria revealed downregulated expression of genes involved in antioxidant defence systems, antioxidant mitochondrial systems, anti-apoptotic markers, necrosis markers and proapoptotic marker BAX, indicating that cerium oxide nanozymes involved in maintaining the redox balance in hMSCs [69].

### In vivo studies

Several studies have reported investigating the biosafety and therapeutic efficacies of nanoceria in tumour models. Fernández-Varo et al. demonstrated that the antioxidative and anti-inflammatory properties of a 4–5 nm nanoceria synthesised by the co-precipitation method can partially revert cell mechanisms involved in tumour progression and significantly improved survival in Wistar rats bearing hepatocellular carcinoma (HCC). Decreased macrophage infiltration, and decreased levels of phosphorylated extracellular signal-regulated kinase 1/2 (P-ERK1/2) related to stress-responsive Ras/MAPK signalling pathway were reported. Nanoceria also restored fatty acids metabolism that was found to be dysregulated in HCC animals as highly proliferative cancer cells demonstrate strong lipid and cholesterol activity to support proliferation [86]. Levels of malondialdehyde MDA (end-product of lipid peroxidation), myeloperoxidase MPO activity (lysosomal oxidising agent) and nitric oxide (inflammatory marker), that were significantly increased in Wistar rats bearing breast tumours, were ameliorated in rats treated with nanoceria [82]. The exact mechanisms in which nanoceria is affecting lipid and cholesterol metabolisms require further study and could provide insights into potential applications of nanoceria in other diseases that arise form dysregulated lipid and cholesterol activities.

The cancer therapeutic potential of nanoceria was investigated in preclinical disease models. Tian et al. reported 51.1% shrinking of tumour weight with intravenously administered porous cerium oxide nanorod prepared via the hydrothermal method and the anti-cancer effect of nanoceria was enhanced to 96.1% with sodium polystyrene sulfonate coating [66]. Intraperitoneally administration of 0.5 mg/kg of a ~ 32 nm nanoceria with negative surface charge (− 26.3 mV) suppressed the growth of WEHI164 tumour [81]. A copper-doped nanoceria showed near complete inhibition of tumour growth (98.5% inhibition) [59]. Tumour growth was inhibited as indicated by the significant down regulation of proliferation marker Ki67 of MDA-MB-231 tumour in mice [59] and HCC in rats [86]. Hematoxylin and eosin (H&E) staining revealed a larger density of necrotic and apoptotic cells in treated tumour tissue sections in contrast to the control group [59, 66, 68]. Terminal deoxynucleotidyl transferase dUTP nick end-labeling (TUNEL) staining of tumour tissue sections showed a higher proportion of DNA damage in nanoceria-treated tumours, suggesting enhanced tumour cell apoptosis [59, 81]. Apoptotic marker genes pro-apoptotic Bax and caspase-3 that are involved in the apoptotic pathway were significantly increased in nanoceria-treated tumours whilst expression of anti-apoptotic Bcl2 was downregulated [66, 81]. Elevated signal from superoxide marker dihydroethidium (DHE) staining of tumour tissues [59, 66, 81] also demonstrated increased ROS production in vivo upon nanoceria treatment to trigger oxidative stress-induced cell apoptosis.

In summary, a considerable amount of research outputs from in vitro and in vivo studies had inferred that nanoceria of various surface characteristics and functionalisation is capable of attenuating tumour growth through inducing programmed cell death in preclinical models and the efficacy is significantly enhanced with surface modifications of nanoceria. With the large variety of methodologies for surface coatings and functionalisation available in the preparation of nanomaterials [52, 53, 87], there is an urgent need in developing a clinically relevant pipeline in the rational design of surface modifications on nanoceria to accelerate the development of potent nanoceria formulations, ensure that the nanoparticles prepared are fit for need and to identify biomolecules as robust, specific biomarkers for monitoring responses to nanoceria administration.

## Nanoceria in metastatic diseases

Activating invasion and metastasis being the hallmark of cancer have been responsible for the majority of cancer-related deaths as the complexity of metastatic progression poses hurdles in the development of efficacious therapeutic approaches, resulting in poor prognosis in advanced cancers [88–90]. Whilst still in the nascent stage of studies, the anti-angiogenic effect of nanoceria could potentially play a role in tumour growth suppression as angiogenesis, another cancer hallmark, is known to be involved in promoting tumour vascularisation and metastasis [91]. Giri et al. first reported in 2013 that treatment with Ce^3+^ dominating nanoceria of ~ 38 nm hydrodynamic radius inhibited migration and invasion of ovarian cancer SKOV3 cells through inhibition of growth factors including stromal cell-derived factor 1 SDF1, heparin-binding EGF-like growth factor HB-EGF and vascular endothelial growth factor VEGF [92]. Metastatic nodules were significantly reduced in size and decreased in numbers in the lungs of nanoceria-treated mice. Tumour xenograft slides were stained with TUNEL to detect cells undergoing apoptosis and CD31 to identify endothelial cells in microvessels. Co-localisation of stains was observed suggesting that nanoceria could contribute to anti-angiogenic effects by inducing apoptosis of endothelial cells in microvessels. Xiao et al. then demonstrated that nanoceria prepared by the thermal decomposition method with 3 nm core size and -18 mV surface charge could suppress the metastatic potential of gastric cancer cells BGC823 and MKN28 [93]. Cancer cells were pre-treated with or without nanoceria before being injected intraperitoneally into nude mice every 3 days. Less metastatic tumours were observed in nanoceria-treated group than there were in the control mice injected with cancer cells that were not co-cultured with nanoceria [93]. Hao et al. treated Herceptin-resistant Pool2-nGL tumours implanted into the mammary fat pads of the mice with nanoceria plus Herceptin. The researchers observed significantly decreased expression of Ki67, VEGF and CD31, suggesting that the combination treatment could suppress tumour growth and metastasis [77]. Recently, Yong et al. reported that nanoceria with a particle diameter of 5–6 nm decreased the expression of hypoxic angiogenic genes HIF-1α and VEGF-A in human melanoma Me1007 cells [40]. VEGF is upregulated by HIF-1α during hypoxia that induces the development of blood vessels in solid tumours and promotes intravasation of cancer cells from primary tumour sites [94].

Zuo et al. established an experimental metastasis model by intravenous injection of melanoma B16F10 cells into tail vein of C57BL/6 mice at day 5 after subcutaneous injection of the melanoma cells [32]. Treatment with a nanoceria-based nanoplatform (IR-780 and metformin-loaded mesoporous silica nanoparticles with nanoceria as gatekeepers) reduced the number of metastatic nodules on the lung surface in comparison to the control group. Expression of N-cadherin was decreased whereas E-cadherin was increased in the tumour tissues exposed to nanoparticles indicating anti-metastatic potential of the nanoplatform [32]. Cadherins are involved in the epithelial-to-mesenchymal transition (EMT) which enhanced the mobility and invasion capabilities of cancer cells that elevated N-cadherin level is reported to promote metastatic behaviour of tumour cells [95] whilst expression of E-cadherin is believed to suppress tumour growth and metastasis [96]. Infiltration of myeloid-derived suppressor cells (MDSCs) in tumour sites under hypoxia via the HIF-1α pathway [97] was significantly reduced in treated tumours [32]. The reduction in MDSC recruitment resulted in a decreased inhibitory effect on T cells and downregulated immune checkpoint programmed death ligand 1 PD-L1 expression [32] which could reverse MDSC-mediated immunosuppression to enhance therapeutic outcomes of immunotherapy treatment and inhibit MDSC involvement in the formation of premetastatic niches, tumour angiogenesis and tumour cell invasion [98]. Zhu et al. investigated a ruthenium-loaded cerium oxide yolk shell nanozymes Ru@CeO_2_-RBT/Res-DPEG for dual chemotherapy/photothermal therapy in orthotopic CT26 colorectal cancer model [99]. The combined treatment resulted in significantly reduced metastasis in intestine with no detectable metastases in liver, spleen, and lung when all mice in the control group has extensive metastases observed.

Liu et al. proposed the use of a homologous targeted nanoceria integrated with dendritic mesoporous silica nanoparticles as neoadjuvant chemotherapy to inhibit metastases of breast cancer without significant systemic toxicity [100]. In vitro wound-healing assay, Transwell invasion assay and increased expression of DHX15 that inhibit the intrinsic invasive and metastatic ability of cancer cells [101] revealed the metastatic inhibitory effect of engineered nanoceria in 4T1 cells [100]. Liu also demonstrated the nanoceria prepared effectively hindered the transdifferentiation of fibroblast to cancer-associated fibroblast (CAF) and reprogrammed CAF back to a normal fibroblast both in vitro and in vivo [100]. CAF is shown to modulate cancer metastasis and has thus attracted great interests as a therapeutic target [102, 103]. Whilst the nanoparticles alone did not exert anti-cancer effects on the primary tumour, pre-treatment with intravenous injection of 1 mg/kg nanoceria coupled with 3 mg/kg doxorubicin (DOX) every 3 days for 4 times resulted in effective tumour growth control. Primary tumours were removed 25 days post inoculation and another 3 times of treatment were administrated as post-surgical management. The number of lung metastasis and liver metastasis modules observed were significantly reduced in nanoceria treated orthotopic 4T1 breast cancer model [100].

In contrast to reported metastatic suppressive effects, naïve nanoceria has also been shown to not exert effective anti-metastatic activity but instead work synergistically with other bioactive drugs. Naz revealed that metastatic lung cancer A549 cells migrated from the invasion chamber to the lower feeder upon treatment with nanoceria in comparison to nanoceria incorporated with lactonic sophorolipids and ganetespib that dramatically decreased the migratory ability of the cells [104].

In summary, nanoceria potentially inhibits metastatic progression in different stages through decreasing cell survival and proliferation, attenuating expression of molecules that are associated with epithelial-to-mesenchymal transition, angiogenesis and vascularisation, or alternatively as neo-adjuvant or adjuvant treatment to sensitise the tumour cells for enhanced therapeutic efficacies of conventional systemic therapies. Further studies could consider the potential of nanoceria as maintenance therapy upon primary treatment to manage the risk of developing metastatic disease.

## Sensitisation of cancer cells for therapies

Apart of direct inhibition of primary tumour growth, nanoceria also assists in combatting drug/treatment resistance through sensitisation of cancer cells to enhance treatment responses. Nanoceria with the ability to generate molecular oxygen in TME is widely exploited as sensitising agents in photodynamic, photothermal and radiation therapies (Table 2). The incorporation of nanoceria with conventional drugs also demonstrated the potential to rectify resistance in targeted and chemo-therapies.

**Table 2:** Summary of nanoceria-based nanoplatform engineered to sensitise tumour cells for enhanced photodynamic PDT, photothermal PTT and radiation therapeutic effects

Nanoplatform	Sensitiser	Targeting moieties on NPs	Cancer cell line	In vitro	In vivo and ex vivo	Treatment protocol
CeO_x_@fMIL [67]	CeO_x_	Labelled with folic acid FA to target folate receptors	HeLa	Signal of cell-permeable hypoxia indicator significantly reduced Strong singlet oxygen sensor green signal generated upon laser irradiation	–	Irradiation with 660 nm laser (200 mW/cm^2^) for 5 min, 6 h post treatment (in vitro)
ICG@PEI-PBA-HA/CeO_2_ [79]	Indocyanine Green ICG Ce^3+^/Ce^4+^ ratio: 0.85	Hyaluronic Acid HA conjugation to target CD44 receptor	MCF-7	Intracellular ROS increased Cancer cell killing enhanced with ICG-CeO_2_ + laser compared to ICG + laser	Blood oxygen saturation increased from ~ 30% basal level to ~ 80% 24 h after IV injection Tumour hypoxia signals significantly reduced Tumours completely destroyed after treatment with nanomaterials and PTT, no sign of recurrence Severe tumour tissue apoptosis observed with TUNEL staining	100 μg/mL ICG in 100 μL injected intravenously into MCF-7 tumour-bearing mice Irradiation with 808 nm laser (1 W/cm^2^) for 4 min, 24 h post-injection (in vivo)
CeO_2_@DNA-DOX [107]	Cy5	DNA sequence complementary to target miRNA-21	HePG2	In absence of laser, intracellular H_2_O_2_ cleared by CeO_2_ to produce O_2_ After NIR illumination, weak endogenous PDT ability of Cy5 enhanced with increasing production of highly toxic ·OH from accumulated O_2_	–	Irradiation with 625 nm laser for 30 min-1 h (in vitro)
CeO_2_@MSNs@IR780/Met [32]	IR780	No targeting ligand, redox responsive CeO_2_ as gatekeepers for release of drugs at H_2_O_2_ rich tumour site	Murine melanoma B16F10 cells	Significant toxic singlet oxygen generated by IR780 from simultaneous generation and economisation of O_2_	H&E staining of treated tumours demonstrated cell integrity destroyed with massive apoptotic bodies Decreased number of Ki67-positive cells indicating tumour cell proliferation inhibited TUNEL staining revealed distinct apoptosis in treated tumour tissues Survival rate of mice extended from 0% in control group to 75% in treated group Metastatic mouse model demonstrated reduced numbers of metastatic nodules on lung surface in treated mice Decreased expression of N-cadherin that promotes tumour metastasis and increased expression of E-cadherin that is anti-metastatic Tumour oxygenated (strong oxygenated haemoglobin signal) Ex vivo HIF-1α staining, protein and mRNA levels of HIF-1α in tumour tissues were all decreased in the treated group	16 mg/kg Met, 20 mg/kg IR780 injected into B16F10 tumour-bearing mice Irradiation with 808 nm laser (1 W/cm^2^) for 5 min (in vitro); 10 min, 8 h post-injection (in vivo)
CeO_2_-PEG-Ce6-GOx (CPCG) [108]	Chlorin e6 Ce6	–	HeLa	Laser-induced cytotoxicity greater than free Ce6 and CPC (NP without glucose oxidase GOx) Large amount of ROS produced Significant cytotoxicity upon laser irradiation	Negligible systemic toxicity PDT/starvation treatment group demonstrated the most significant tumour growth inhibition	1.5 mg/mL CPCG in 100 μL injected intratumourally into HeLa tumour-bearing mice Irradiation with 660 nm laser (0.2 W/cm^2^) for 6 min (in vivo)
CeO_x_-EGPLGVRGK-PPa [58]	Pyropheophorbide-a PPa Ce^3+^/Ce^4+^ ratio: 0.25	EGPLGVRGK substrate peptide of matrix metalloproteinase-2 (MMP-2)	HePG2	Nanoprobe endocytosed, peptide cleaved by endogenous MMP-2 to release PPa Large amount of ROS produced upon laser irradiation Dose-dependent cytotoxicity under irradiation indicating excellent PDT efficacy	–	Irradiation with 660 nm laser for 5 min (in vitro)
ATP-HCNPs@Ce6 [117]	Ce6 Ce^3+^/Ce^4+^ ratio: 1.7:1	–	Murine mammary carcinoma cells 4T1	ATP improved stability and biocompatibility of NPs Co-localisation of NPs and lysosomes Enhanced PDT phototoxicity Higher intracellular oxygen generated in presence of H_2_O_2_	80% tumour volume reduction in ATP-HCNPs@Ce6 + PDT H&E staining revealed larger areas of cell death and inflammatory infiltration in treated tumour tissues H&E staining showed no significant damage to vital organs	50 µM Ce6 in 20 µL injected intratumourally into 4T1 tumour-bearing mice Irradiation with 660 nm laser (0.05 or 0.2 W/cm^2^) for 5 min, 2 h post-injection (in vivo)
MCSCe [110]	Carbon nanosphere (CS) Ce^3+^/Ce^4+^ ratio: 0.96	4T1 cancer cell membrane coating for homotypic targeting	4T1	SOD-like activity catalysed transformation of intracellular superoxide into H_2_O_2_ Accumulated intracellular H_2_O_2_ decomposed to ·OH through NIR-activated thermal catalysis Homologous targeting, higher uptake and enhanced NIR irradiation-induced cytotoxicity in cancer cells MTMP declined and mitochondrial dysfunction indicative of cell apoptosis	Preferential accumulation of nanomaterials at tumour sites Local hyperthermia with temperature increased to ca. 55 °C upon irradiation Improved therapeutic effect with nanoceria formulated treatment (MCSCe + laser) compared to carbon nanosphere (MCS + laser) Significant apoptosis/necrosis observed in H&E and TUNEL stained tumour tissues from treated group ~ 90% injection dose cleared in 1 week H&E staining, liver and kidney function markers, serum biochemical and haematological parameters of treated groups all were similar to the control group and fell within normal ranges	20 mg/kg MCSCe in 100 µL PBS injected intravenously into 4T1 tumour-bearing mice Irradiation with 808 nm laser (1.5 W/cm^2^) for 8 min, 24 h post-injection (in vivo)
NCeO_2_-PEI-MoS_2_ [111]	Molybdenum sulphide MoS_2_	–	MDA-MB-231	Intracellular ROS generation upon laser treatment increased as a function of nanoceria concentration up to 0.5 mg/mL > 90% cytotoxicity under laser light irradiation at 0.25 and 0.5 mg/mL nanoceria	–	Irradiation with 808 nm laser (0.5 W/cm^2^) for 5 min (in vitro)
CNPs@(g-C_3_N_4_/CeO_x_)-Met [112]	g-C_3_N_4_	–	HepG2	NPs slowly penetrated into the center of 3D spheroid tumour cell model and released g-C_3_N_4_ after 4 h Temperature rose and remained above 42 °C for PTT Complete killing of cells with synergistic PTT/PDT Enhanced ROS production that depleted GSH to reduce oxidation resistance against PDT procedure	Temperature rose to 42.1 °C and photothermal conversion ability remained stable after 5 cycles of laser irradiation PET imaging revealed alleviation of tumour hypoxia by NPs, not by oxygen supply from blood vessels Significant inhibition of tumour growth and longer survival period with combined PTT/PDT Higher degree of necrosis and apoptosis observed from H&E staining of treated tumour tissues Intensity of cell proliferative marker Ki67 and hypoxia marker H1F-1α signals declined No significant H&E pathological variations observed in vital organs of treated mice in comparison to the control group Haematology assays indicated no acute toxicity to mice	5 mg/mL nanomaterials in 100 µL injected intravenously into HepG2 tumour-bearing mice Irradiation with 808 nm laser (0.5 W/cm^2^) for 10 min (in vitro); 8 min for PTT, 15 min for PTT/PDT, 12 h post-injection (in vivo)
Bi_2_S_3_@Ce6–CeO_2_ [113]	Bi_2_S_3_ (PTT) Ce6 (PDT)	–	4T1	Enhanced ROS production with CeO_2_ formulated NPs Intracellular H_2_O_2_ consumed by CeO_2_ to produce cytotoxic ^1^O_2_ PDT/PTT combination treatment nearly ablated all cancer cells Real-time cellular analysis revealed effective PDT/PTT effects in inhibiting cancer cell growth	Tumour temperature rose to 60.6 °C upon injection and irradiation, but no significant temperature change in other tissues PDT or PTT alone inhibited tumour growth to some extend Near complete tumour eradication with PDT/PTT combination treatment TUNEL staining revealed most extensive necrosis in the NPs + PDT/PTT treatment group Immunohistochemistry staining showed NPs + PDT/PTT treatment damaged CD31-positive tumour microvessels and inhibited number of Ki67-positive proliferating cells	2 mg/mL NPs in 50 µL injected intratumourally into 4T1 tumour-bearing mice Irradiation with 660 nm laser (0.2 W/cm^2^) for 2 min or 808 nm laser (1.5 W/cm^2^) for 3 min
GDY–CeO_2_–miR181a–PEG–iRGD [78]	CeO_2_ and miR181a	iRGD peptide that mediates binding to neuropilin-1 receptor upon proteolytic activation	Human esophageal cancer cells ESCC (KYSE30 and KYSE180)	HIF-1α protein level in KYSE30 cells decreased gradually under normoxia and hypoxia Radiation-induced DNA damage and apoptosis enhanced in KYSE30 and KYSE180 cells	Radiosensitivity enhanced in KYSE30 tumour-bearing models and patient-derived xenograft models evident by the significant inhibition in tumour growth	Intravenous injection of nanocomposites to ESCC patient-derived xenograft model 6 or 12 Gy X-ray radiation (repeat injection + irradiation) after injection

### Photodynamic therapy

Photodynamic therapy (PDT) being a promising non-invasive adjuvant anti-cancer treatment requires photosensitisers that transform light energy from an irradiation source into other forms that kill cancer cells. PDT effectively destroys tumours by energising photosensitisers at a specific wavelength, which triggers photochemical reaction utilising endogenous molecular oxygen (O_2_) as substrate in the production of cytotoxic ROS molecules [75, 106]. However, the limited photodynamic reaction of photosensitisers have been reported due to the O_2_ deprivation at tumour sites [67, 79]. Thus, nanoceria-based therapeutics with the capability to catalyse O_2_ accumulation within cancerous tissues are developed as effective photosensitising agents.

**Fig. 3:**
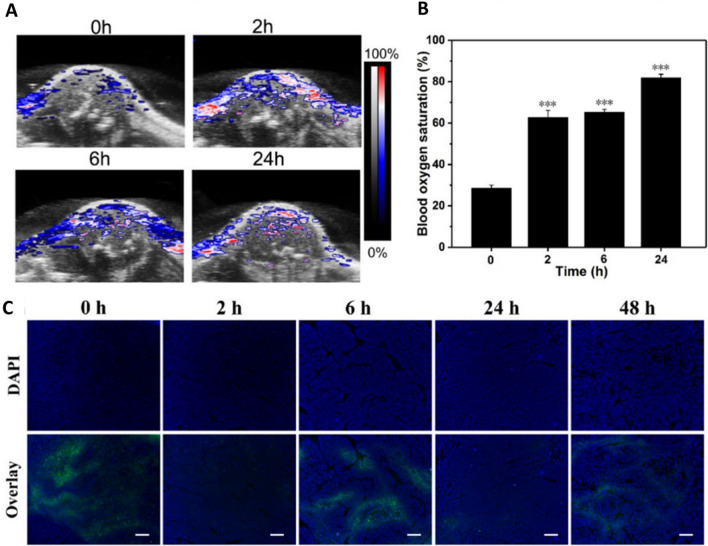
MCF-7 tumour-bearing BALB/c-nude mice injected with 100 μL of ICG@PEI–PBA–HA/CeO_2_ (including 1 mg/mL HA/CeO_2_) through the tail vein. **a** Photoacoustic images of tumour blood oxygen saturation levels and **b** average saturation levels shown by photoacoustic images at different times after intravenous injections demonstrated significant improvement in blood oxygen saturation level in hypoxic tumours. **c** Representative tumour hypoxia immunofluorescence images revealed substantially reduced tumour hypoxia signals after administration of ICG@PEI–PBA–HA/CeO_2_ (the scale bar is 50 μm). Reprinted with permission from[79]. Copyright 2020 American Chemical Society

Liu’s group reported a functionalised nanoceria in a metal–organic framework acting as PDT sensitiser, inducing apoptosis in hypoxic tumour cells upon laser irradiation through a tandem homogenous catalysis process: decomposition of H_2_O_2_ to O_2_ relieving hypoxia followed by conversion of O_2_ to cytotoxic ROS. Additionally, the nanosystem was engineered with Cy3-labelled peptide which was cleaved by caspase-3 activated in apoptotic events, achieving in situ monitoring of therapeutic response [67]. A CeO_2_ nanozyme system loaded with photosensitiser indocyanine green (ICG) improved the killing of MCF-7 cells in comparison to free ICG as CeO_2_ increased intracellular ROS that induced cell death, in addition to the thermal effects of ICG upon laser irradiation. Blood oxygen saturation level was significantly improved, and hypoxia signal was substantially reduced in tumours, suggesting that the CeO_2_ NPs acted as artificial enzymes catalysing the production of O_2_ from H_2_O_2_. Ex vivo analyses revealed severe tissue apoptosis and complete disappearance of tumours without recurrence in any location (Fig. 3) [79]. Zhang et al. reported a CeO_2_ nanoclusters loaded with weak PDT photosensitiser Cy5 that with near-infrared (NIR) illumination, Cy5 + CeO_2_ demonstrated higher cytotoxicity in hepatic carcinoma cells than Cy5 alone [107]. This indicated that the decomposition of H_2_O_2_ to O_2_ by CeO_2_ became the raw material and photochemical reaction driving force for Cy5 to convert O_2_ to ·OH radicals.

Zuo et al. successfully synthesised a mesoporous silica nanoparticles (MSNs) loaded with photosensitiser IR780, mitochondrial respiration inhibitor Metformin (Met) and CeO_2_ NPs (CeO_2_@MSNs@IR780/Met) [32]. The nanoplatform promoted tumour oxygenation through CeO_2_’s O_2_ generation and Met’s O_2_ economisation, enhanced IR780’s capability of generating toxic singlet oxygen from molecular oxygen. It is worthy to note that alleviating tumour hypoxia also attenuated the recruitment of myeloid-derived suppressor cells which mediated immunosuppression and contributed to low response to antitumour immunotherapy [32, 74]. A nanocomposite consisted of PEG-functionalised CeO_2_, photosensitiser chlorin e6 (Ce6) and glucose oxidase (GOx) was synthesised for enhanced PDT through sequential catalytic reactions involving dual-path modulation of H_2_O_2_. CeO_2_ transformed superoxide anion into H_2_O_2_ and GOx decomposed glucose into H_2_O_2_. CeO_2_ then catalysed the generation of O_2_ from the accumulated H_2_O_2_. As glucose consumption by GOx requires O_2_, the O_2_ supplied by CeO_2_ enhanced the glucose depletion that impeded nutrient supply and starved the tumour cells for a synergistic photodynamic/starvation treatment [108]. Whilst authors claimed that NPs resulted in significant tumour inhibition, this study did not perform any histological examination of extracted tumours nor further analysis of biomarkers. This study only looked at the relative tumour volume to assess therapy efficacy and did not include vital information such as the frequency of NP administration.

Fan et al. successfully engineered a stimulus-responsive cerium oxide-based nanoprobe (CeO_x_-EGPLGVRGK-PPa) that is selectively activated in cancer cells for efficient PDT. The octahedral nanoceria with Ce^3+^/Ce^4+^ of 0.1/0.9 demonstrated a more effective catalase-mimicking activity relieving tumour hypoxia. Nanoceria with a wide absorption spectrum from 300 to 1200 nm was utilised as the acceptor of the Förster resonance energy transfer (FRET) effect that quenched the PPa’s fluorescence during the “silent state”. Matrix metalloproteinase-2 (MMP-2) overexpressed in cancer cells will cleave the peptide EGPLGVRGK off to activate the nanoprobe at the tumour site, resulting in PPa restoring its fluorescence and producing toxic singlet oxygen ^1^O_2_ upon 660 nm light irradiation [58].

### Photothermal therapy

Photothermal therapy (PTT) is another promising phototherapy that utilises photosensitisers to convert NIR light into heat energy, creating localised elevated temperature that kill tumour cells through cancer cell membrane destruction, tumoral DNA denaturation and blocking of angiogenesis. However, PTT faces drawbacks with insufficient heat generation at disease sites and thermal damage to surrounding tissues [75, 109]. Thus, nanozymes like nanoceria are incorporated into the design of PTT nanomaterials for targeted local hyperthermia and to protect normal tissues from collateral damage.

Zhang synthesised a cerium-dotted carbon nanosphere (MCSCe) that utilised nanoceria with Ce^3+^/Ce^4+^ ratio of 0.49/0.51 for its SOD-like activity to decompose superoxide to H_2_O_2_. The carbon nanosphere formed hyperthermia upon NIR irradiation, which then catalysed the production of ·OH radicals from H_2_O_2_ under heat stress [110]. Murugan reported a molybdenum sulphide nanoflakes (MoS_2_) decorated with nanoceria that improved the photoconversion efficiencies of the photothermal agent MoS_2_. When irradiated with laser light, bare MoS_2_ flakes showed a temperature increase of up to 41.8 °C, whilst MoS_2_ flakes decorated with 0.25 and 0.5 mg/mL nanoceria enhanced the heat generation ability, with temperature increase up to 53.7 and 47.6 °C, respectively. These nanoceria-MoS_2_ formulations also showed enhanced photostability with consistent heating–cooling profiles of the nanoflakes when irradiated repeatedly for five laser on/off cycles [111].

### Photodynamic/photothermal therapy

Jiang et al. reported multifunctional, H_2_O_2_-sensitive and O_2_ self-regenerative UCGM NPs (CNPs@(g-C_3_N_4_/CeO_x_)-Met) as a potential PDT/PTT agent. Catalase-mimicking nanoceria oxidised H_2_O_2_ into O_2_ and Met inhibiting cellular respiration alleviated the hypoxic condition in deep tumour tissues. Results attained demonstrated no significant changes in the blood vessels of tumour-bearing mice thus indicating that the tumour hypoxia was relieved by the administration of NP. Accumulated O_2_ then detached photosensitiser g-C_3_N_4_ that penetrates to the core of the tumour tissue. Upon exposure of 808 m laser, the NPs with upconversion ability will emit short wavelength laser, exciting g-C_3_N_4_ to generate toxic ROS. Additionally, UCGM NPs displayed excellent performances in upconversion luminescence (UCL), MR and CT imaging that can be exploited for imaging-guided drug delivery system[112]. Zeng et al*.* synthesised Bi_2_S_3_@Ce6–CeO_2_ with Bi_2_S_3_ as PTT agent and as photoacoustic (PA) and computed tomography (CT) contrast agent, Ce6 as a photosensitiser for PDT and CeO_2_ as O_2_ evolving-agent. CeO_2_ converted H_2_O_2_ into O_2_ which then was converted to toxic singlet oxygen through Ce6-mediated photodynamic reaction. The NPs itself did not exert any toxic effects both in vitro and in vivo, indicating excellent biocompatibility and biosafety. However, upon irradiation with 660 nm (PDT) or 808 nm (PTT) laser alone, significant anti-cancer effects were observed. The synergistic combination of PDT followed by PTT demonstrated near complete eradication of tumour [113].

### Radiation therapy

Zhou et al*.* designed a 2D graphdiyne (GDY) immobilised ultrasmall CeO_2_ nanozymes, loaded with microRNAs (GDY–CeO_2_–miR181a–PEG–iRGD) to overcome tumour radioresistance and enhance radiotherapy efficacy in oesophageal squamous cell carcinoma. Nanoceria relieving tumour hypoxia and miR181a directly targeting RAD17 (checkpoint protein for DNA repair, upregulated in radioresistant cells) acted as radiosensitisers, enhanced the intracellular radiation energy deposition which resulted in DNA damage of cancer cells [78].

### Targeted therapy

Hao et al. proposed the use of nanoceria to sensitise Herceptin-resistant HER2 + breast cancer cells to Herceptin therapy, which is a monoclonal antibody targeting epidermal growth factor receptor 2 (HER2) proto-oncogene found overexpressed in 20–25% of breast tumours [77, 114]. While the employment of nanoceria alone did not demonstrate significant killing of cancer cells in vitro, Herceptin and nanoceria together exhibited an enhanced inhibition on survival and proliferation of Herceptin-resistant cells under hypoxia than under normoxia. Hence, nanoceria potently blocked the induction of HIF-1α to VEGF signalling pathway under hypoxia which is associated with aggressive tumour progression and metastasis [77, 115].

### Chemotherapy

Wu et al. discovered that pre-treating lung cancer A549 cells with low dose (10 μg/mL) hydrothermally prepared nanoceria exhibiting uniform sheet-like shapes (50 nm thickness) enhanced the anti-cancer efficacy of chemotherapeutic agent DOX through the disruption of mitochondrial function and impairment of DOX detoxification. Nanoceria triggered ROS production which severely depolarised mitochondrial membrane and consumed intracellular GSH that typically initiated drug detoxification. Nanoceria pre-treated cells demonstrated depletion of ATP required to support drug pump activity, resulting in the restricted efflux of DOX (31.3% vs 59.3% drug loss in pre-treated vs non-treated cells) [116].

## Synergistic anticancer effects

Nanoceria in combination with other anti-cancer agents, have shown to demonstrate synergistic cancer-killing activities. Wang et al. reported a Ce doped Cu–Al layered double hydroxide ultrathin nanosheets loaded with photothermal agent ICG as an integrated synergistic chemodynamic (CDT) and photothermal (PTT) therapeutic agent. The nanocomposite first catalysed H_2_O_2_ decomposition to ·OH radicals, and in the presence of oxidised cerium and copper ions, depleted intracellular reducing agent GSH which is a potent ·OH scavenging agent. The GSH-reduced metal ions catalysed further Fenton reaction generating toxic radicals, enhancing CDT efficacy. The production of cytotoxic ROS was boosted by hyperthermia from laser irradiation of ICG-loaded nanocomposite that the combination index (CI) was determined to be less than 0.8 (Synergism: CI < 1 [118]). Advantaging from its strong NIR absorption and paramagnetic Cu(II), the nanocomposite showed excellent dose-dependent photoacoustic (PA) and magnetic resonance (MR) imaging performance [60]. Dong and group synthesised a cerium-based nanozyme (PEG/Ce-Bi@DMSN) with PTT capability. Near-infrared illumination resulting in local hyperthermia enhanced the POD- and CAT-mimicking catalytic activities, and GSH consumption of nanoceria. Significantly stronger anti-cancer effects and higher survival rate were achieved with PEG/Ce-Bi@DMSN + laser in U14 tumour-bearing mice. The presence of element Bi in the nanocomposite also allowed in vivo high-contrast computed tomography (CT) imaging [119].

## Nanocarrier to improve drug delivery

Advantaging of the nanoscale size, nanoceria has been used in cancer treatment as a nanocarrier to enhance the bioavailability of anticancer agents by improving their solubility, stability and circulating half-life. Chen et al*.* fabricated a CeO_2_@SiO_2_-PEG nanoplatform as a potential drug delivery carrier for both hydrophilic and hydrophobic drugs [120]. Naturally derived bioactive polyphenols curcumin and quercetin complexed with cerium significantly decreased the viability of MDA-MB-231 and A375 cells upon blue light irradiation in comparison to free compounds. Significant changes in cell morphology with increased programmed cell death indicated that cerium complexation could overcome the low solubility of curcumin and rapid elimination of quercetin which then enhances their PDT capability [121]. Zholobak synthesised a nanoceria-curcumin composite that significantly reduced the autoxidative degradation rate and inhibited UV-induced photodegradation of curcumin through the inactivation of ROS. When treated with nanoceria-curcumin and UVC (253.7 nm) irradiation, enhanced cytotoxic effects were observed in tumour cells whilst normal cells were protected against the short-wave UV irradiation [122]. Nanoceria functionalised with targeting ligand also allows targeted delivery of two or more drugs for enhanced therapeutic effects. Naz et al. reported a folate-conjugated nanoceria coloaded with potential anticancer drug lactonic sophorolipid (LSL) and Hsp90 inhibitor ganetespib (GT). The nanodrug demonstrated a higher affinity and synergistic cytotoxicity for lung carcinoma A549 cells, whilst sparing non-disease CHO cells [104].

**Fig. 4:**
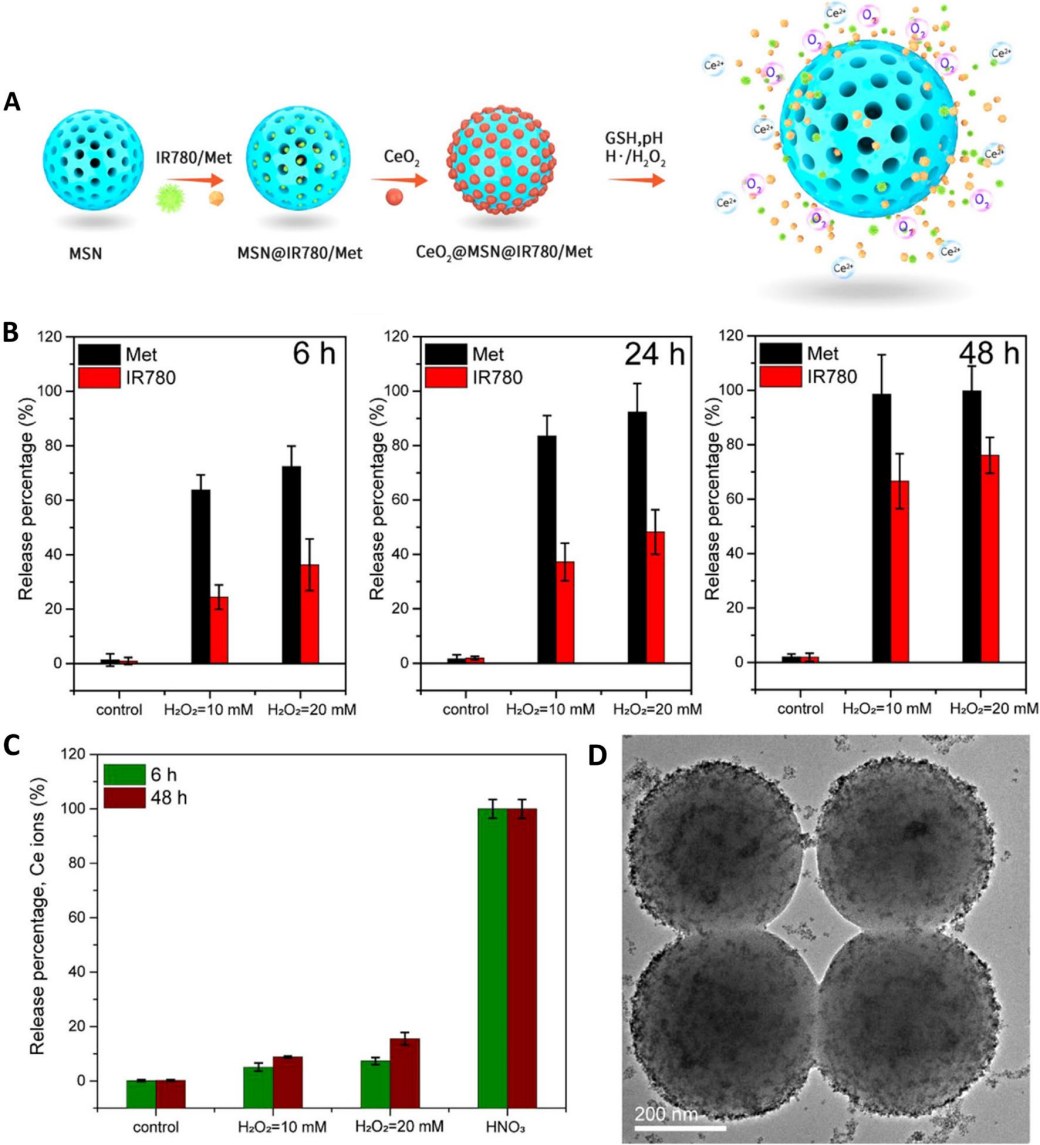
H_2_O_2_ responsive nature of nanoceria exploited for tumour-selected drug delivery. **a** Working principle of CeO_2_@MSNs@IR780/Met NPs that were synthesized by loading photosensitiser IR780 and mitochondrial respiration inhibitor Metformin into mesoporous silica nanoparticles MSNs with CeO_2_ as the gatekeepers. IR780 and Met were released, while O_2_ and Ce^2+^ were generated after the etching of CeO_2_ NPs in response to the special tumour microenvironment with lower pH and excessive H_2_O_2_. **b**, **c** In vitro release behaviour detection: **b** Time-dependant release performance of IR780 (red column) and Met (black column) from the CeO_2_@MSNs@IR780/Met NPs in the absence and presence of 1.0 and 2.0 M H_2_O_2_. **c** Etching performance of Ce NPs. Ce ions detected by inductively coupled plasma (ICP) analysis under various conditions: control, 10–20 mM H_2_O_2_, and nitric acid. **d** TEM image of the CeO_2_@MSNs@IR780/Me nanocomposition in 20 mM H_2_O_2_ solution after 6 h. Reprinted with permission from [32]. Copyright 2020 American Chemical Society

H_2_O_2_ responsive nature of nanoceria has been exploited for tumour-selective drug delivery (Fig. 4). Controlled release of drugs (IR780 and Metformin) loaded on mesoporous silica NPs (MSN) reservoirs was achieved by employing CeO_2_ (~ 4 nm) NPs as gatekeepers sealing the channels (~ 2 nm pore size). CeO_2_ slowly etched with excessive H_2_O_2_ in TME, eventually detaching from the pores and releasing the loaded drugs into tumour sites. Plasma half-life of the nanoplatform was ~ 22.5 h with almost double amount of IR780 accumulated in tumour tissues, which was a striking improvement from the ~ 10.8 h half-life of free IR780 [32]. Singh reported the use of amine functionalised nanoceria to cap the pores of mesoporous silica loaded with the chemotherapeutic agent doxorubicin. The nanoplatform demonstrated a typical drug release profile in an acidic medium (release % pH 4 > pH 5 > pH 6) as the NH_2_-nanoceria was protonated and detached from the mesoporous silica; on the other hand, DOX release was negligible in a neutral medium (pH 7.4) preventing pre-mature release of drugs under physiological conditions [123]. Sedighi developed an amine functionalised MSNs capped with nanoceria for controlled delivery of tyrosine kinase inhibitors (TKIs) sorafenib (SFN) or sunitinib (SUN) for the treatment of HCC. The nanoceria capping of MSNs potentially decreased protein adsorption that improved the colloidal stability of NPs under physiological conditions. The redox-sensitive nanoceria etched from the nanocomposite under exposure to GSH that is elevated in tumour cells to release loaded anti-cancer agents [124].

Whilst an increasing number of studies had proposed the use of nanoceria as a drug delivery agent also harbouring anti-cancer activities, Fu reported that the SOD and CAT-like activities of nanoceria are only significant at concentrations higher than 10 µg/mL [54]. Thus, future studies must ensure that the formulations are carefully tuned whereby nanoceria and loaded drugs are delivered at doses with the highest efficacies and minimal toxicities. Researchers should also clarify the concentrations of each component in the proposed materials instead of only reporting the loaded drug or concentration of whole nanoparticles, this could help with characterising the effects of different modifications on the nanozymes.

## Protection against treatment-induced cellular damage

The sustained release of radical species from radiation therapy leads to genetic damage and disrupts bone repair and regeneration, resulting in bone loss and susceptibility to bone fracture in cancer patients exposed to ionising radiation [125, 126]. Wei et al*.* reported a Ce^3+^ dominating nanoceria (60% Ce^3+^ on the surface of nanoceria) as a potential multifaceted regulator to protect human bone marrow-derived stem cells from ionising radiation-induced cell damage. Pre-treating cells with nanoceria significantly reduced irradiation-induced ROS generation, attenuated nuclei leakage and DNA fragmentation. Elevated expression of tumour suppressor gene p53, osteopontin, osteocalcin and bone morphogenetic protein 2; increased number of autophagic vacuole and reduced unwanted cell senescence were observed. Replenishment of NPs to the stem cells post irradiation helped to achieve optimum osteogenic differentiation [126]. Kadivar investigated the potential radioprotection and radio mitigating effect of nanoceria purchased from US Research Nanomaterials, Inc. (10–30 nm) in rats’ lungs exposed to 18 Gy whole-thorax X-ray. Nanoceria administered before or after radiation treatment both demonstrated improvements in the radiation-induced histopathological changes in lung tissues [127].

Chemotherapy involving repeated systemic administration of aggressive anti-cancer drugs is associated with detrimental effects on the survival and growth of healthy cells [128–135]. For this reason, nanoceria-based materials have been engineered to protect or relieve normal tissues from the anti-cancer agents-induced toxicity. A doxorubicin (DOX) loaded, Cu-doped nanoceria enhanced the targeted tumour suppression effect of DOX whilst protecting normal cells from DOX-induced oxidative stress through its antioxidant capacity [59]. The hepatic and kidney functions and heart damage indicators of the treated groups were all in the normal range indicating excellent biosafety of this DOX-loaded nanoceria [59]_._ Nanoceria as a promising antioxidant and anti-inflammatory agent is also used to ameliorate chemotherapeutic agent cisplatin-induced nephrotoxicity. Swiss mice pre-treated with 2 mg/kg nanoceria (287.6 nm hydrodynamic diameter) prior to cisplatin induction showed significant improvement in general health evident by the restored body and kidney weight [136]. Reduction in levels of renal injury markers (creatinine and blood urea nitrogen), oxidative stress marker malondialdehyde and pro-inflammatory cytokines were reported [136]. Renal injury including tubular dilation and lesions found in cisplatin control groups were absent in nanoceria-treated groups [136]. All parameters and biomarkers assessed in animals that received daily injections of nanoceria for the whole duration of the experiment, were consistent with healthy control groups (isotonic saline injections), indicating the biocompatibility and safety of nanoceria [136].

These promising results further established nanoceria as an innovative treatment approach as there are higher risks of noncancer-caused deaths in diagnosed cancer patients contributed by treatment complications and infections [137]. Thus, nanomaterials with the capability of treating cancers, inhibiting metastatic progression, exerting protective effects on healthy tissues, and relieving adverse events of conventional therapies could improve patient prognosis and quality of life upon administration of treatment.

## Effects on healthy cells and normal tissues

Nanoceria-based therapeutic agents have been shown to selectively deplete or generate cytotoxic ROS depending on the pH of the microenvironment. Acidic cancerous tissues were killed but normal tissues at neutral pH were spared as nanoceria acted as ROS scavenger, producing non-toxic H_2_O and O_2_ [68]. Tian et al*.* reported that the oxidative activity of porous CeO_2_ nanorods and sodium polystyrene sulfonate (PN–CeO_2_–PSS) was inert in neutral media, hence little to no interference on healthy tissues was observed [66]. The enzymatic properties of nanoceria are also redox-sensitive that can be engineered to selectively catalyse reactions in cancerous tissues. A ceria nanozyme (ATP-HCNPs@Ce6) was reported to be an efficient PDT agent only under the presence of high-level H_2_O_2_ which is characteristic of cancer cells, whilst demonstrating a protective effect on normal cells [117]. Localisation studies revealed that a dextran-coated gadolinium-doped nanoceria Ce_0.9_Gd_0.1_O_1.95_ did not penetrate cell nuclei and comet assay confirmed that the NPs did not exhibit genotoxic effects in either normal or cancer cells [69].

As it is widely known that nanoceria favours ROS production under acidic conditions, normal cells might be damaged if nanoceria is internalised and localised in highly acidic lysosomes (pH 4.5–5.0) [138]. Upon cell internalisation, a nanoceria encapsulated in a metal–organic framework core–shell nanohybrid was found to be localised in acidic lysosomes in HeLa cells and HaCaT cells [67]. Thus, it is crucial that all nanoceria-based NPs are engineered with targeting and/or protecting moieties to minimise accidental uptake by normal cells [68]. Cancer cell membrane (CCM) was utilised to camouflage the nanoparticles for homotypic binding towards cancer cells and as a carrier to prevent premature drug leakage that might produce side effects in normal tissues [49, 68]. Enhanced uptake of CCM-coated nanoparticles by its source cancer cells [59] and excellent tumour growth suppression (cancer inhibition ratio 92.8%) with the weight of mice remained stable across the two weeks period [68] were reported. Ex vivo studies showed that a large amount of non-coated NPs deposited in the kidney and liver, while CCM-coated NPs accumulated in tumour [110]. Conjugation of ligands targeting cancer biomarkers onto nanoceria is also widely used to enhance cancer cell uptake and to mitigate accidental uptake of the nanomedicine by normal cells. Modifications of nanoceria with HA [79, 85] and FA [67] targeting CD44 receptor and folate receptor respectively that are known to be overexpressed in cancerous cells were reported. Babu Varukattu performed a competition assay by pre-treating triple-negative breast cancer cells with free HA before treatment with HA-conjugated nanoceria (HA-CePEI-NPs) and showed a significant reduction in NPs uptake, indicating that the NPs were internalised via receptor-mediated endocytosis [85]. However, the incorporation of targeting moieties would potentially affect the therapeutic efficacy of nanoceria as the concentration of surface oxygen vacancies would be compromised. Thus, Ma et al. demonstrated targeted killing of tumour cells by engineering nanoceria of various morphologies and surface oxygen vacancies (nanoceria-rod, nanoceria-poly and nanoceria-cube) through altering ratios of sodium hydroxide and temperature of the hydrothermal reaction utilised to prepare the material. Nanoceria-rod with the highest oxygen vacancies among the three synthesised nanoceria was characterised to have an isoelectric point (IEP) of 6.2 that remained positively charged in the acidic TME (IEP > pH_tumour_) but negatively charged in the neutral environment (IEP < pH_normal_). Under the acidic TME, the nanoceria-rod selectively entered tumour cells and distributed in lysosomes and phagosomes to produce ROS and induce cell apoptosis. In contrast, the small amount of nanoceria that entered normal cells stayed in the cytoplasm with no cytotoxicity effects reported [51].

In terms of hemocompatibility, no haemolytic activity was detected in red blood cells treated with dextran-coated nanoceria Ce_0.9_Gd_0.1_O_1.95_ revealing potential for intravenous administration [69]. It is worthy to note that the hemocompatibility of nanoceria requires individual assessments as it varies with the characteristics and surface functionalisation of the nanoparticles [139]. Bare Cu-doped nanoceria showed slight haemolysis (4.09%) at 1000 μg/mL but the haemolysis ratio decreased to 1.62% when coated with a cancer cell membrane [59]. No significant haemolysis was observed in red blood cells collected from healthy mice and exposed to 1 mg/mL nanorod nanoceria [66].

Different methodologies were reported suggesting that nanoceria exerts no acute toxicity in animal models during treatment. H&E staining results indicated no significant morphological changes in tissues of major organs including heart, liver, spleen, lung, and kidney harvested from animals administered with nanoceria-based treatment [32, 66, 68]. Blood routine examinations including levels of red blood cells, hemoglobin, platelets, and white blood cell count were all in the normal reference ranges [59, 66, 112]. The particles at nano-scaled size were mainly non-specifically distributed in the liver, kidney, and spleen [44, 66, 68, 140] thus effects towards hepatic and renal function need to be critically assessed. Liver function biomarkers examined from the blood of treated animals including total, protein, total bilirubin, albumin, alanine aminotransferase, aspartate aminotransferase and alkaline phosphatase were reported to be in the normal ranges [32, 59, 66, 81, 112]. No significant differences in kidney function parameters including blood urea nitrogen, creatinine, glucose levels were reported comparing nanoceria-treated animals to the control group [59, 66, 112]. Cerium was also detected in the urine of treated mice, suggesting that nanoceria can be metabolised and removed from the body through a normal excretory system [66].

It is worthy to note that the majority of research outputs reported no significant toxicities that were based on in vitro cell viability assays and in vivo adverse events that, in fact, are not capable of reproducing the tumour microenvironment [141]. In contrast to the reported excellent biocompatibility of nanoceria designed to exert cancer-killing effects, nanoceria exposure has been found to potentially induce health complications. Ballesteros reported that nanoceria demonstrated carcinogenicity in human bronchial epithelial cells BEAS-2, with tumoral effects enhanced when combined with cigarette-smoke condensate exposure [142]. The proportion of cells exhibiting cancer stem cells-like features and expression of microRNAs related to carcinogenic process were increased upon nanoceria and co-exposure treatments. Maternal exposure to nanoceria also potentially resulted in pregnancy complications [143]. Zhong et al. injected 5 mg/kg nanoceria prepared with microemulsion method intravenously to pregnant BALB/c mice [144]. Whilst no damage was observed in vital organs in mother mice, fewer pups and lighter pup weights were observed compared to the control group. Uterine bleeding, severe uneven embryo development and foetal resorption rate were higher for nanoceria-treated group. Further study from the group revealed that trophoblast dysfunction mediated by excessive autophagy activation through the mTORC1 signalling pathway following nanoceria exposure led to impairment in placental development [145]. Hence, it is vital that the engineering of nanoceria platform requires the incorporation of biocompatible materials and targeting ligands to not only enhance efficacy but also to minimise potential damage to vital organs. Furthermore, the methodologies in validating biosafety of nanoceria must be thoroughly evaluated so researchers would not risk overlooking the complex biological reaction in the response of the human body to nanoparticles administration.

## Challenges in clinical translation

Despite promising findings in pre-clinical studies, there is still no nanoceria study registered on the clinical trials database (https://www.clinicaltrials.gov) for applications in human diseases. The limited progression towards clinical translation could be due to the drawbacks in conventional methods and the absence of standardised protocols to comprehensively characterise the surface properties, catalytic activities, and biosafety of nanoceria [146–148]. Liu and group had worked on demonstrating the use of radar analysis to assess the enzyme-mimicking activities of nanoceria prepared with varying synthesis parameters to guide the rational development and selection of a desired nanozyme [149]. However, the group only assessed differences in synthesising temperature that a lot more work would be required before a general consensus could be met. Furthermore, research laboratories generally reported characterisation of the established nanomaterial whereby the bioactive properties could not be precisely pinpointed to nanoceria itself, it could potentially be contributed by other active components or as a result of synergistic effects. Characterising nanoceria-based materials without functionalisation is not always possible due to the stability and solubility of bare cerium oxide in aqueous solutions [150]. As the correlation between surface characteristics and bioactive properties of nanoceria could not be effectively established, it becomes challenging in developing novel strategies to design and tune nanoceria-based platform to achieve desired therapeutic effects. There are lacking of studies on the interactions between nanoceria and endogenous biomolecules in physiological-relevant disease models. This resulted in the sustainability and specificity of nanoceria’s ROS modulating activities in the biological systems in comparison to its natural enzymes counterparts remains unclear [87, 151]. This poses multiple challenges in accelerating bench-to-bedside as there are no established guidelines available to identify nanoceria formulation that would be effective against specific diseases and no biomarkers to stratify patient groups that would benefit from nanoceria treatment.

Discrepancies in the toxicity assessments reported also raised concerns over the safe use of nanoceria. It has been demonstrated that nanoceria does not exert genotoxicity [69, 152] but Arslan and Akbaba reported that cerium oxide in both micro and nano sizes was genotoxic on human lymphocyte cells even at low concentration (0.78 ppm) [147]. Furthermore, due to difficulties in accessing high-performance imaging modalities for pH measurement in disease models [153, 154], findings of nanoceria exerting pH-responsive catalytic effects in vivo are indirectly inferred from other methodologies such as pO_2_ level and hypoxia signals. Increased level of molecular oxygen, decreased level of hypoxic signals and enhanced efficacies of PDT were exploited to infer that nanoceria catalysed accumulation of O_2_, relieving tumour hypoxia at disease sites.

Apart from that, the biocompatibility and toxicity studies of nanoceria are generally short-term effects examined in cell lines and mouse models lacking tumour heterogeneity which is a hallmark of human tumour progression and impacts clinical outcomes [155]. García-Salvador et al. revealed minor toxicity in cultured cells after acute exposures (24-h treatment) of nanoceria with a hydrodynamic size of 44.13 nm and surface charge of + 36.16 mV synthesised by the conventional gel-sol process. However cytotoxic and inflammatory responses were observed in the more physiologically relevant human airway epithelial model after subchronic exposure up to 90 days [156]. Tentschert and group performed a 2-year study on the low-dose 28.4 nm nanoceria exposure via inhalation in Wistar rats and demonstrated exposure dose and time-dependent CeO_2_ burden on lung and lymph nodes in close proximity of the lung [157]. Lee et al. on the other hand reported no general systemic toxicity to Sprague–Dawley rats and no reproductive and developmental toxicity to their pups after repeated oral exposure to nanoceria following testing guideline from the Organization for Economic Co-operation and Development (OECD) Working Party on Manufactured Nanomaterials [158].

The contradictions in safety assessments are likely resulting from the large variety of possible modifications during the preparation of nanoceria particles. The toxicity profile of nanoceria is therefore yet to be fully attributed. In addition to concerns over toxicology, the therapeutic potential of nanoceria in metastatic disease which is the leading cause of poor prognosis is also insufficiently studied. The reported in vivo anti-metastatic effects of nanoceria are deduced from the number and size of metastatic nodules forming from primary tumours at specific timepoints. There are unmet needs in investigating the therapeutic effects on the already developed metastases, long-term disease progression and survival. Thus, to accelerate the clinical translation of nanoceria, researchers should validate the safety of potential nanomedicine candidates in humans and justify that the therapeutic benefits would outweigh the possible adverse effects.

## Conclusions and future perspectives

Nanoceria-based therapeutics in pre-clinical studies focused on resolving tumour hypoxia, which is the hallmark of TME positively correlated with poor prognosis. Overconsumption of O_2_ by the rapid proliferation of tumour cells leads to chemotherapy tolerance and insensitivity of PDT and RT. Advantaging from the excessive H_2_O_2_ produced by tumour cells, CeO_2_ NPs generate continuous supply of oxygen at the tumour site, resolving the hypoxic TME for effective therapies. The regenerative valency states, enzyme-like activities, and redox-modulatory capabilities of nanoceria presents it as a therapeutic candidate targeting the aberrant redox status in tumorous tissues. Since the valency states are self-regeneratable, treatment can potentially be given at lower or less frequent doses. The pH-responsive, dual-catalytic nature of nanoceria also protects normal tissues from oxidative stress and treatment-induced cellular damage. Interestingly, there is no comprehensive study on the correlation of Ce^3+/^Ce^4+^ ratio and the anti-cancer effects of nanoceria in vitro and in vivo. Also, many reported studies did not include this vital parameter which makes comparison between studies complicated. Henceforth, further investigations must be performed in the future to completely elucidate the effect of Ce^3+^/Ce^4+^ ratio on the therapeutic efficacy and toxicity of cerium-based NPs. The exact redox pathways and mechanisms affected by nanoceria in biological systems are also yet to be fully elucidated as research has been focused on the preclinical therapeutic efficacy but often lacks metabolic activities, pharmacokinetics and pharmacodynamics of the nanomaterials. The non-redox bioactivity of nanoceria has also been suggested proposing nanoceria’s anticancer effects are contributed by additional non-redox mechanisms [48]. Future research should comprehensively characterise synthesised nanoceria to establish the redox and non-redox physicochemical properties of the NPs under physiological conditions and disease-specific circumstances. Metabolic fate and long-term effects of nanoceria in biological systems need to be fully assessed to ensure efficacy and safety for clinical applications. Whilst the majority of the published in vivo studies displayed therapeutic efficacy in subcutaneous tumour models, this does not reflect the true manifestation and complexity of cancer in patients. Nonetheless, this is the case in not only nanoceria-based NPs but in many preclinical anti-cancer studies whereby subcutaneous tumours are the model of choice. Hence, care must also be taken when developing animal models and investigating the potential therapeutic effects of nanoceria. The disease models should be developed to be able to represent the complexity and heterogenic nature of cancers so that studies are more clinically relevant. With thorough verification of the therapeutic effects, drug delivery efficacy, biological fate and biosafety, nanoceria-based system could serve as a promising approach to managing cancers and other ROS-related diseases. The nanomaterials that are carefully engineered with excellent biocompatibility can potentially bypass undesirable effects that arise with conventional treatments and improve clinical outcomes and quality of life for cancer patients.

## Data Availability

Not applicable.
